# Understanding Plain English summaries. A comparison of two approaches to improve the quality of Plain English summaries in research reports

**DOI:** 10.1186/s40900-017-0064-0

**Published:** 2017-10-09

**Authors:** Emma Kirkpatrick, Wendy Gaisford, Elaine Williams, Elizabeth Brindley, Doreen Tembo, David Wright

**Affiliations:** 1 Southampton Clinical Trials Unit, University of Southampton, Southampton, UK; 2 Southampton Health Technology Assessment Centre (SHTAC), University of Southampton, Southampton, UK; 3 National Institute for Health Research Evaluation, Trials and Studies Coordinating Centre (NETSCC), University of Southampton, Southampton, UK

**Keywords:** Plain English summaries (PES), Lay summaries, NIHR, Flesch reading ease, Readability, Communication, Journalology, Patient and public involvement (PPI)

## Abstract

**Plain English summary:**

There is a need for the authors of research reports to be able to communicate their work clearly and effectively to readers who are not familiar with the research area. The National Institute for Health Research (NIHR), along with a number of other funding bodies and journals, require researchers to write short lay summaries, often termed plain English summaries (PESs), to make research accessible to the general public. Because many researchers write using technical, specialised language, particularly in scientific reports, writing PESs can be challenging. In this study we looked at how to improve the quality of PESs. We took PESs which had been submitted to the NIHR Journals Library and asked authors to rewrite them using new guidance. We also asked an independent medical writer to edit the summaries. We measured the quality of these three versions (original summary, rewritten summary and edited summary) in two ways. First, we asked a group of people who were not specialists in the subject area to read and rate how easy the summaries were to understand. Secondly, we used a well-known measure called the Flesch reading ease score to assess how easy the PESs were to read. We found that there was no difference in how easy people found the summaries to understand across the three versions. However, the PESs that were rewritten by the authors and that were edited by the independent medical writer were both easier to read than the originals. This shows that PESs can be improved and for organisations who feel that employing an independent writer to edit summaries, providing clear, practical guidance to authors may be a cost-effective alternative.

**Abstract:**

**Background:**

Plain English summaries (PES) or lay summaries are often included as part of research reports and journal articles. These summaries are vital to ensure that research findings are accessible and available to non-specialist audiences, for example patients and members of the public.

Writing a PES requires the adoption of a different style than is generally used in a traditional scientific report, and researchers can find this challenging. This study explored two possible ways to improve the quality of PESs in the NIHR Journals Library: 1) Providing enhanced guidance to authors and asking them to rewrite the PES and 2) Employing an independent medical writer to edit the PES.

**Methods:**

We compared the three versions of the PES (original, author rewritten and independent writer edited) to assess 1) how easy they were to understand and 2) how easy they were to read. In order to establish how easy PESs were to understand, a group of 60 public reviewers read a set of summaries and rated them on a four point scale from “Did not understand” to “Understood all”. The Flesch reading ease score was used to measure how easy the summaries were to read.

**Results:**

Results indicated no significant difference across the three versions of the PES in terms of ease of understanding. However, both the author rewritten and independent writer edited versions were significantly easier to read than the original. There was no significant difference in ease of reading between these two versions.

**Conclusion:**

These findings suggest that employing independent medical writers to edit PESs and providing clear, practical guidance to authors are two ways in which the readability of PESs could be improved. Results have implications for journal editors and publishers seeking to enhance accessibility and availability of research findings.

**Electronic supplementary material:**

The online version of this article (doi:10.1186/s40900-017-0064-0) contains supplementary material, which is available to authorized users.

## Background

In the last decade, with an increase in open access to biomedical research and greater involvement of patients and public in research, members of the public are being encouraged to be better informed in their treatment choices and to actively engage in research. Many major funding bodies such as the National Institute for Health Research (NIHR), the Medical Research Council (MRC) and research charities require public involvement in study design and management. Members of the public make a valuable contribution to clinical trials and medical research, ensuring that studies are relevant to patients. To maximise the impact of public input, it is essential for this population to be able to understand the content of written reports and therefore for researchers to communicate scientific information in an understandable and accessible manner.

To ensure medical and scientific information, in particular results of clinical trials, is disseminated in an accessible way, medical research organisations and open access journals require researchers to provide a summary of research proposals and findings in a form that is understandable to the general public. These summaries, which are termed plain English summaries (PESs) or lay summaries, are required in addition to the standard scientific content [1]. In 2013, alongside the launch of the open access NIHR Journals Library, it was stipulated that all NIHR-funded projects required PES as part of the final report published in the journals library. Historically, however, the guidance provided to help researchers was broad and not prescriptive. More detailed instruction has been provided to guide researchers in the production of PESs for grant applications (a requirement for NIHR funding since 2014) as part of the “*make it clear campaign*” promoted by INVOLVE, the national advisory group that supports active public involvement in NHS, public health and social care research [2]. Similarly, the National Cancer Research Institute (NCRI) Clinical and Translational Radiotherapy research working group consumer members produced excellent guidance on how to write lay summaries for proposals guidance meetings [3]. These guidance documents are specifically geared towards prospective PESs, i.e. for planned research. However, PESs for research reports, which summarise the findings of a research project, require a slightly different focus.

The ability to understand the written word and text has long been a subject for exploration and research by educationalists and writers and has been assessed in various ways. Readability has been defined by several measures, including the length and complexity of words and sentence structure, as well as how frequently common words are used. A number of formulae, based on these measures, have been developed to predict ease of reading [4] such as the Flesch reading ease (FRE) score [5] and the Fog score [6]. These scores are widely used by editors and writers to ensure that articles and stories are pitched at an appropriate level for the target audience, for example within education, for government documents and newspapers [7]. The FRE score, which is considered one of the oldest and most accurate readability formulae [8], is incorporated into Microsoft Word software. It calculates ease of reading based on length of words and sentences and generates a score between 0 and 100 where higher scores indicate that the text is easier to read. Broadsheet newspapers [9] and introductory student textbooks [8] typically have FRE scores of around 50–60, whereas academic papers are generally in the range of 20–40. FRE scores of 50 or greater are recommended for effective communication with non-subject-specialists [2, 8, 10]. The FRE score, however, is recognised as a crude test which rewards the use of short sentences and short words (containing fewer syllables) and does not account for coherence or layout of text [4, 11]. In addition, it does not offer any information about how easy the text is to understand. Small modifications to a text can improve FRE scores but changes in isolation can be counterproductive and lead to text that is more difficult to understand [12].

It is known that academics, who deal with technical terminology and jargon on a daily basis, can struggle to communicate their research in simple terms [13]. With many researchers believing that the use of specialised language is essential to ensure accurate communication of research findings, non-scientists can struggle to understand articles written by these researchers [2]. A number of studies have aimed to identify the essential factors that help non-specialists understand the content of scientific and medical research reports. These studies highlight the need to write in short, clear sentences, reduce jargon, and use analogies to communicate complex ideas as necessary [14]. Other factors include the use of subheadings to help break up text, ensuring logical structure and flow, as well as encouraging the writer to read the text aloud and to ask friends and colleagues to read the text and provide feedback [2, 15].

Whilst many organisations requiring a PES provide guidance to researchers on how to write one, the level of guidance varies [16, 17]. Some simply provide a paragraph stating that summaries should be suitable for people without a specialist scientific or medical background and therefore researchers should avoid scientific/ technical terms or acronyms [18], whereas others may describe more fully the general outline required [15, 19] or provide website links to advisory documents [20]. Recommended advisory documents include those produced by the Plain English Campaign [21], the NIHR “make it clear campaign” [22], research on clear writing techniques [6, 10] and university-produced guidance [23]. It is also recognised even when guidance is provided, it is not always clear and comprehensive and website links may not be up to date or easy to navigate [24]. As a result, researchers may continue to struggle to produce understandable summaries and effectively assess their quality [2, 25].

In recognition of the challenges faced by researchers writing PESs, a number of medical research charities and open access journals, such as Cancer Research UK (CRUK) and Public Library of Science, employ dedicated teams of specialist writers to produce easy-to-understand PESs [17]. Whilst CRUK is highly regarded for its high quality PESs, the considerable time and cost involved in employing specialist writers makes this model infeasible for many organisations [25]. During a consultation of open access stakeholders on attitudes towards PESs, it was concluded that there was a lack of consensus about the most cost-effective way of producing PESs and further work investigating different approaches to producing and funding these summaries was recommended [26]. A report commissioned by the NIHR INVOLVE in 2013 evaluated guidance for assessing and writing PESs used when reviewing funding applications and to provide a publicly available, stand-alone summary of research. The report highlighted that guidelines are often not clear enough and recommended that further, clearer advice and guidelines should be provided to authors to enable them to both write and assess PESs more effectively [2].

## Aim

The aim of this study was to assess two possible methods of improving ease of reading and understanding of PESs published in the NIHR Journals Library: 1) Providing enhanced guidance to authors and asking them to rewrite the PES and 2) Employing an independent medical writer to edit the PES.

The results of this study will inform best practice for producing accessible PESs for the NIHR Journals Library in the future.

## Methods

### Participants

#### Summary authors

Participants were authors of research reports covering a range of projects funded by four NIHR research programs: Health Technology Assessment (HTA), Efficacy and Mechanism Evaluation (EME), Health Services and Delivery Research (HS&DR) and Public Health Research (PHR). Authors were approached by the research co-ordinator and invited to take part in this study.

#### Independent medical writer

An independent medical writer with a BSc Hons degree, with experience of providing writing and editing services to the pharmaceutical and healthcare industries was employed to edit PESs.

#### Raters of summaries

Summaries were assessed by sixty participants from NETSCC’s panel of public reviewers. This panel consists of approximately 700 adults, who have registered an interest in reviewing commissioning briefs or research proposals on behalf of the NIHR. Reviewers are drawn from a cross-section of the public who express an interest in medical research and development for either personal reasons or general interest but do not usually have professional expertise. Reviewers were approached by the research co-ordinator and offered the opportunity to take part in this research study.

These reviewers were made aware of and understood the confidential nature of the summaries, which contained results of unpublished research.

### New author guidelines

Guidelines for producing PESs were re-written to include more practical advice and instruction for authors (Additional file 1)*.* They were based on outcomes from the *Patients Participate!* initiative [25] and the report on *Improving the quality of plain English summaries for National Institute for Health Research (NIHR) funded research* [2].

### NIHR journals library reports

Forty PESs were obtained from research reports resulting from NIHR-funded research which were submitted to the NIHR journals library over a seven month period from March to September 2015. Summaries covered a range of health-related topics, written and submitted by academic researchers across four of the research programmes of the NIHR: HTA, EME, HS&DR and PHR. The decision to include 40 PESs was a pragmatic consideration to allow data collection in a reasonable timeframe.

### Design

We conducted a repeated-measures prospective cohort study with two interventions. The first intervention involved authors revising the PES in line with updated guidance (Additional file 1). The second intervention involved an independent medical writer editing the PES. Ethics approval was granted by the Faculty of Medicine Ethics Committee, University of Southampton (Submission Number 13372).

### Outcomes

#### Ease of understanding rating

A group of reviewers representative of the target audience was asked to read a set of PESs supplied alongside the title of the research report and rate how easy they were to understand.

First, participants were asked, *how familiar were you with the subject of this text prior to reading?* Response options, presented horizontally, were: 1 - No prior knowledge, 2 – Some knowledge from newspapers, television etc., 3 - I have personal experience of this subject, 4 – I have professional experience of this subject, 5 - other, please specify in the free text box provided. Participants were not limited to a single response on this question.

The second question was, *how well do you feel you understood the main message of this text?* Four possible response options were presented horizontally: 1 - Did not understand, 2 - Understood some, 3 - Understood most, 4 - Understood all. Participants were limited to a single response but also had the option to provide additional comments in a free text box.

#### Flesch reading ease score

The FRE score was used to measure ease of reading of the PESs. It is generated with the following formula:

$$ 206.835-1.015\ \left(\frac{total words}{total sentences}\right)-84.6\left(\frac{total syllables}{total words}\right) $$

This score is easily generated on a computer and gives an indication of how easy a document is to read. Scores are generally in the range of 0–100, with higher scores indicating more readable text. The Independent and Times newspapers have FRE scores of about 50 [27]. Table 1 summarises level of readability corresponding to score ranges. The advantages of this tool are its ease of use and objectivity.

**Table 1:** Interpretation of Flesch reading ease scores [8]

FRE Score	Ease of reading	Examples of text location
<30	Very difficult	Scientific Journal
30–50	Difficult	Academically orientated magazine
50–60	Fairly difficult	Broadsheet paper/introductory textbooks
60–70	Standard	Tabloid magazine/ beauty magazine
70–80	Fairly easy	Science fiction

### Procedure

**Fig. 1:**
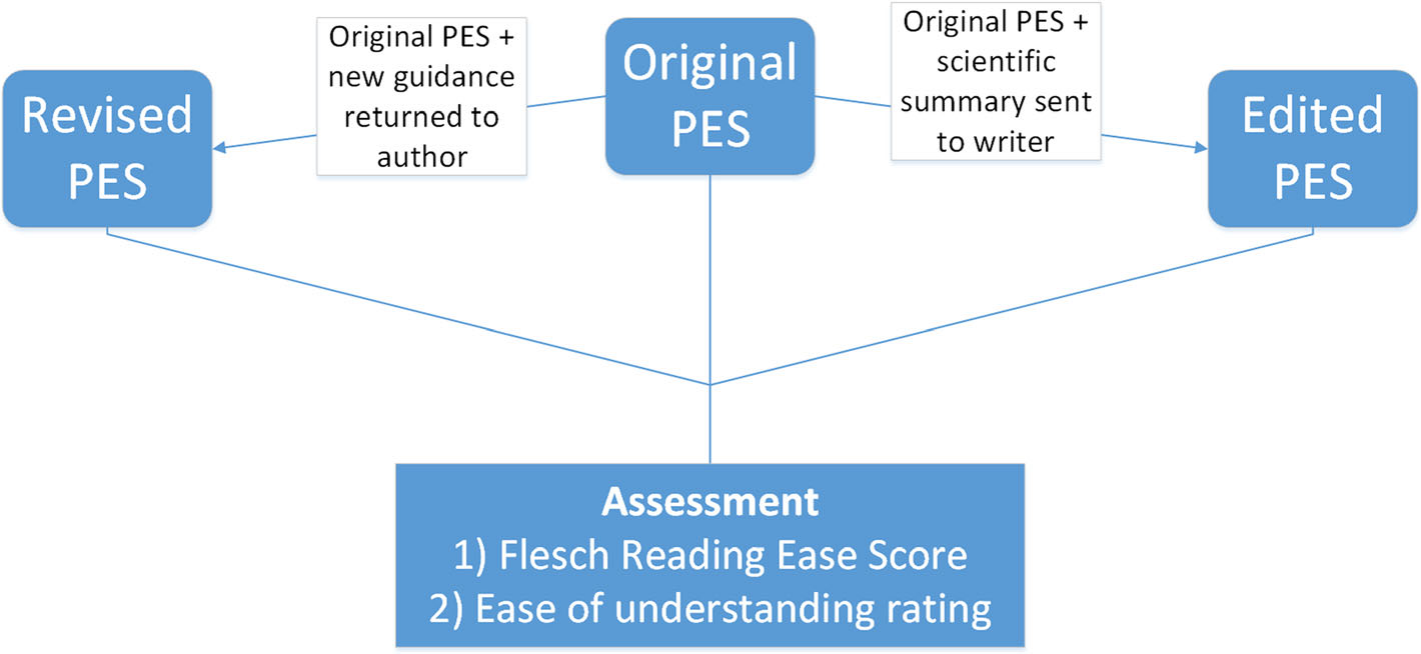
Schematic diagram outlining the production of PES versions from the original PES and methods used for evaluation of ease of reading and understanding. Each PES was rated three times

Report authors were asked to revise the submitted PES in line with new guidance (Additional file 1). The originally submitted summaries were also sent to an independent medical writer, alongside the corresponding scientific summaries (up to 2400 words in length), to edit (Fig. 1).

Members of NETSCC’s public reviewer panel were then contacted by email and invited to take part in this study. Reviewers were told that the quality of PESs in the NIHR Journals Library was being evaluated; however, they were not told that the two interventions were being evaluated, in case this inadvertently affected their ratings. Reviewers were remunerated at the standard reviewer rate for the NIHR.

The first sixty people to respond were included in the study and were assigned an alphanumeric ID code which was used as an identifier for the study. A link to a consent form and online survey was sent to participants. Participants consented to keep information contained within the PESs confidential as many of the reports had not yet been published.

Each participant read and rated six PESs - two original, two author revised and two edited by an independent medical writer. Participants were never presented with more than one version of the same PES. Each PES was rated by three participants. The task took approximately 30 min to complete but no time constraints were imposed. Participants could read at their own pace and take breaks if they wished. PESs were presented in a box on the screen, underneath which the two questions were presented. For each summary, participants could edit their response until they pressed the *save and continue* button, after which the next summary appeared and no return to the previous PES was possible.

### Analysis

#### Ease of understanding ratings

A Fisher’s exact test was used to determine whether the distribution of ratings of understanding were significantly different between the groups. Comparisons were original vs. author revised; original vs. independent medical writer edited; author revised vs. independent medical writer edited. Analysis was performed in Stata with a significance threshold of 0.05.

#### Flesch Reading Ease score

FRE scores were calculated for all summaries and scores for each version (original vs. author-revised vs. independent writer-edited) were compared with a Friedman test. Wilcoxon signed-rank tests with Bonferroni correction were used to explore these differences. Statistical analysis was performed with SPSS software [28] and results were deemed significant if *P* values were less than 0.05.

A sensitivity analysis was performed using the Gunning Fog Index [29] using the same statistical tests as FRE score.

$$ 0.4\ \left[\left(\frac{\mathrm{words}}{\mathrm{sentences}}\right)+100\left(\frac{\mathrm{complex}\  \mathrm{words}}{\mathrm{words}}\right)\right] $$

## Results

### Ease of understanding

**Fig. 2:**
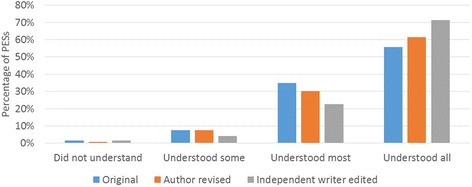
Distribution of understanding ratings across PES versions

Figure 2 shows the distribution of understanding ratings for each version. The Fisher’s exact test showed that edited summaries were not scored significantly differently to original summaries (*p* = 0.06). Specifically, for example, although a lower proportion of summaries were rated as ‘understood all’ for original compared to edited summaries (55.8% vs. 71.7%), and ratings of understood some and understood most were higher in the original summaries compared to edited, the distribution of the understanding ratings was not significantly different.

Similarly, author revised summaries were not rated significantly differently to original summaries (*p* = 0.81), despite more of the revised summaries being rated as ‘understood all’ than the original versions (60.7% vs. 55.8%).

The third comparison between author revised and independently edited summaries showed no significant difference in ease of understanding rating (*p* = 0.22), though a higher percentage of the independently edited summaries were rated as ‘understood all’ (71.7% vs. 61%).

### Ease of reading

FRE scores were recorded for the original, author-revised and independently edited PES (Fig. 2). Statistical analysis revealed that both the author-revised and independent-writer edited summaries had higher FRE scores than the originally submitted versions (*p* < 0.001). However, no significant difference was observed between scores for the author-revised and the independent writer edited versions (*p* = 0.12).

A sensitivity analysis with the Gunning Fog Index as the outcome revealed the same pattern of results. Author-revised and independent-writer edited summaries were both easier to read than the originally submitted versions (*p* < 0.001); while there was no significant difference between the author-revised and independent writer edited versions (*p* = 0.17).

### Familiarity with subject matter

PES raters’ familiarity with the subject matter is summarised in Table 2. Most raters had little to no prior subject knowledge: 167 (46%) had no prior knowledge and 105 (29%) had some knowledge from newspapers and the media.

**Table 2:** Raters’ familiarity with summary topics

Rater prior knowledge of subject area	(n)
1. No Prior Knowledge	167
2. Some knowledge via newspapers and media	105
3. Some knowledge via personal experience	60
4. Some professional knowledge	21
5. Other (vague or non-response)	7

### Free text comments

Comments provided within the additional comments box were coded (blind to version) according to themes emerging from the data. These are summarised in Table 3.

**Table 3:** Themes emerging from qualitative feedback Comments were collated and grouped into positive and negative feedback, and suggestions for further improvements

		Author original (n)	Author revised (n)	Independent writer ﻿edited﻿ (n)
Positive	General positive	34	42	53
	Headings useful	0	17	9
	Terminology explained	3	5	9
Negative	Jargon/terminology	25	19	12
	Title not clear	18	19	23
	Insufficient detail	15	11	20
	Ambiguous language	15	8	12
	Acronyms/abbreviations	8	5	4
	Sentence length/structure	8	8	7
	Grammar	6	7	3
Desirable	Headings	13	5	1
	Less detail	4	0	0
	Conclusion	6	6	6

Overall, many comments were positive. However, a few negative themes also emerged. Representative quotations are included.

Comments, alongside corresponding reader scores for understanding, including;

*“this piece of writing appears to be more complex due to the amount of medical and research terminology” (understood all),*

“*Very clear and easy to understand but I did not know what QALY stood for”* (*understood all*)

and

*“the style of this is a bit pompous and uses a lot of non-plain English jargon”* (understood most ),

re-inforce the importance of reducing technical jargon and complex language.

The use of short sentences was also highlighted as beneficial:

“*liked the shorter sentences - made it clear and easy to understand*” (understood all).

In addition, the use of ambiguous language was frequently mentioned. For example, one participant commented:

*“is discharge to do with phlegm coming out of the lungs or release from hospital?” (understood all),*

or

*“Theoretically clear, but I found myself re-reading certain sentences a number of times to be sure I'd grasped exactly what they were trying to say, and why” (understood all).*

Comments surrounding the need for headings were frequent in the original summary versions. The incorporation of headings, in both author revised and independently edited summaries prompted positive comments, as readers recognised that the structure and reading experience was improved. A comment on an original summary:

“*I do think this would look a little less daunting and be easier to mentally digest if it were not in one paragraph” (understood all)*,

contrasts with

*“Great use of titled paragraphs to keep the piece clear and easily understood”* (understood all)

in a summary edited by the independent medical writer.

Negative comments such as

*“The title is quite intimidating for a lay person” (understood all),*

or

“*bearing in mind that when searching on the internet you tend to get a list of titles, for lay people the title should also be in plain English” (*understood most *)*

and

*“the title really must be put into simpler language otherwise, one can hardly be bothered to read the rest”* (understood most),

suggest that authors should be encouraged to produce simpler titles for PESs to ensure that they are accessible and available, as well as easily searchable to those seeking information online.

## Discussion

This study explored two possible ways to improve the quality of PESs in the NIHR Journals Library: 1) Providing enhanced guidance to authors and asking them to rewrite the PES and 2) Employing an independent medical writer to edit the PES. While no significant differences were observed across versions in terms of ease of understanding, both the author revised and independent writer edited PESs had significantly higher FRE scores than originally submitted PESs, indicating improvements in ease of reading.

The apparent discrepancy between the two outcome measures could be for a variety of reasons. Factors that affect the FRE score are number of words relative to number of sentences (shorter sentences = higher score) and number of syllables relative to number of words (fewer syllables = higher score). However, simply using shorter words and sentences does not always equate to improved understanding. Moreover, changing text to improve FRE score can sometimes have negative impact on understandability [12]. Ease of understanding was measured on a four point scale and scores were negatively skewed, i.e. for most PESs, regardless of version, raters reported that they understood all. This makes it difficult to detect subtle differences in understanding across versions and there was little room for improvement in ratings. It may have been better to use a larger scale or ask people to estimate the proportion of the PES they understood.

Some raters’ comments revealed a degree of confusion despite the raters reporting that they understood all. It is possible that some PESs were easy to read and understand and others were difficult to read or confusing, requiring several attempts to discern the key message. In future studies, limiting or measuring how long raters spend reading PESs could offer some insight into this. There may also be a discrepancy between actual and self-reported understanding. While this may be the case, assessing true understanding is non-trivial. True understanding could be judged by asking a set of questions designed to test the participants’ understanding. However, designing these questions would be difficult, as they would need to test both that the participant understood the whole summary, and that they understood the specific details within it. An alternative way of testing comprehension might be to ask raters to summarise their understanding of the PES. However, this task relies on more than understanding – it tests the rater’s ability to accurately communicate their understanding and introduces an assessor who must determine whether the rater has truly understood the PES. While self-reported understanding may not reflect comprehension it is the most meaningful measure of accessibility to a PES user, e.g. patient or member of the public.

A limitation of the study was the inability to differentiate the effect of the enhanced guidance from the effect of asking authors to revisit their PESs with no competing tasks and where a switch in writing style was not required. It would be interesting to explore whether this improvement is sustained when enhanced guidance is offered as standard practice or whether it is more important to ask authors to revisit or write PESs when the main scientific report has been completed. Future studies should disentangle these factors in order to inform best practice. Only one independent medical writer was employed to edit PESs in this study. It would have been preferable to include a team of writers in the evaluation to make results more robust. In future studies independent medical writers could be asked to write PESs from scratch, rather than editing existing summaries.

Author views should be explored in tandem with assessments of new approaches to producing or editing PESs. A survey by Biomed Central exploring whether researchers felt their research would benefit from a professionally written lay summary revealed that 49% somewhat or strongly agreed that it would, 17% were neutral and 34% somewhat or strongly disagreed [30]. The large minority of authors who are unconvinced of the value of a professionally written PES may be unhappy for someone outside of the research team to write it and may be concerned about a perceived lack of control. The issue of who pays for professionally written PESs also requires attention, as 48% of those surveyed responded that they would not pay for this service. It remains unclear, therefore, how to gain co-operation of researchers in introducing independent medical writers and how this should be funded. For organisations with limited funding, the results of this study suggest that asking researchers to revise their PES in line with clear guidelines may offer a cost-effective alternative to employing and independent medical writer with similar improvements in quality.

Responses to the additional comments box in this study highlighted an ongoing need to reduce jargon and ambiguity. In addition, it was noted that the use of clear sub-headings helped to break up text and improve flow of the PES. Whilst this is supported by previous research [14, 15], this study also highlights the need for clearer, more understandable titles to help readers to find and engage with research reports relevant to their interests. Authors were not asked to provide plain English titles as part of this study, neither has this been within the scope of other guidance documents [10, 22, 23]. However, based on the consistent feedback from participants we suggest that this is something that should be incorporated into PES guidelines in the future.

Whilst researchers do not always consider that communicating science to the general public is part of their role [23], effective scientific communication between patients and public and decision makers is known to increase research impact [31, 32]. Though researchers increasingly recognise the value of PESs, the content and accessibility of information provided by funders to help guide the process of writing a PES is variable [17]. For those who continue to struggle to communicate their research in an accessible way, patients and members of the public may have much to offer, bringing an interested but non-professional viewpoint. By involving this group in the production of a PES, researchers may benefit from insights regarding jargon and specialist terminology ensuring that it is easily understood by non-experts.

## Conclusion

PESs are a valuable tool for accessibly communicating research findings to non-experts. Here we have highlighted two ways to improve the ease of reading of PESs: 1) providing more explicit guidance to authors and asking them to revise PES drafts and 2) employing an independent medical writer to edit PESs. The results of this study are of relevance to journal editors and will inform practice in the NIHR Journals Library.

## Additional files

Additional file 1: Updated guidance provided to authors. (DOCX 35 kb)

Additional file 2: Table of Acronyms. (DOCX 11 kb)
