# Correction to: Understanding Plain English Summaries: A Comparison of two Approaches to Improve the Quality of Plain English Summaries in Research

**DOI:** 10.1186/s40900-017-0082-y

**Published:** 2017-12-18

**Authors:** Emma Kirkpatrick, Wendy Gaisford, Elaine Williams, Elizabeth Brindley, Doreen Tembo, David Wright

**Affiliations:** 1 Southampton Clinical Trials Unit, University of Southampton, Southampton, UK; 2 Southampton Health Technology Assessment Centre (SHTAC), University of Southampton, Southampton, UK; 3 National Institute for Health Research Evaluation, Trials and Studies Coordinating Centre (NETSCC), University of Southampton, Southampton, UK

## Correction

After publication of our article [1] it has come to our attention that Fig. 3 was accidentally omitted. In the section “Ease of reading”, the sentence “FRE scores were recorded for the original, author-revised and independently edited PES (Fig. 2).” should read “FRE scores were recorded for the original, author-revised and independently edited PES (Fig. 3).” Figure 3 and its legend appear below.

**Fig. 3:**
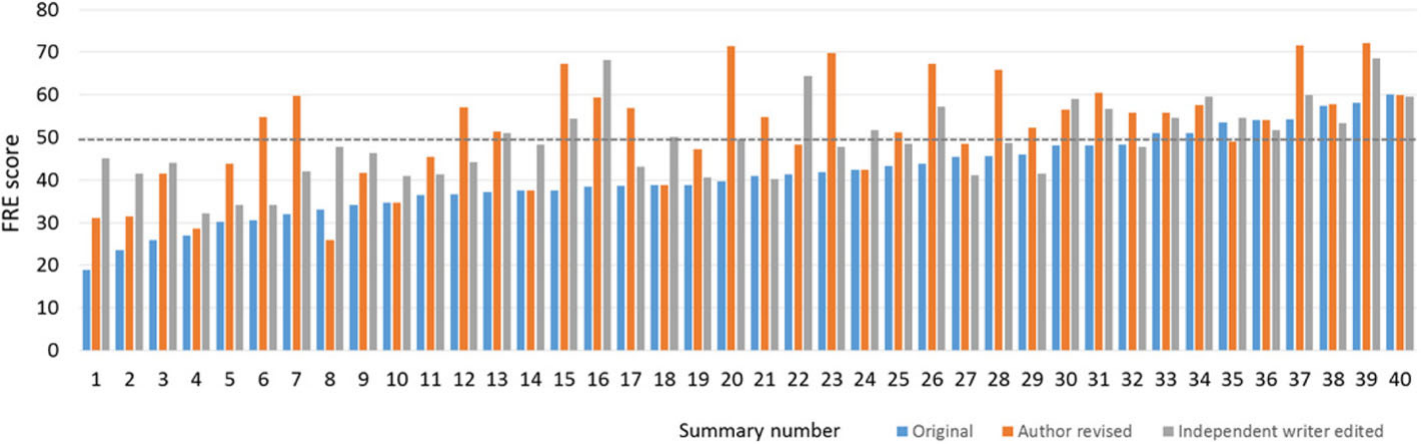
FRE score for summaries compared across three groups. Horizontal dashed line indicates an FRE of 50, which is approximately equivalent to a broadsheet newspaper [27]
