# Supplementary material for: Understanding Plain English summaries. A comparison of two approaches to improve the quality of Plain English summaries in research reports
Source: Res Involv Engagem. 2017 Oct 9;3:17. doi: 10.1186/s40900-017-0064-0 (PMC5632836; doi:10.1186/s40900-017-0064-0)
Supplement: Supplementary file 1 — Updated guidance provided to authors. (DOCX 35 kb) [file 40900_2017_64_MOESM1_ESM.docx]

Additional file 1

Writing a Plain English Summary.

Guidance document

The NIHR is committed to making research available and accessible to the public. Your plain English summary will be published online and may be read by patients and non-experts as well as medical and healthcare professionals. Your summary should be **no longer than 250 words** (excluding headings) and easily understood by someone with no medical or scientific training.

Your summary must be brief and accessible, focusing on the most important aspect of your research - it is not expected to contain the same level of detail as your scientific abstract or summary. You should pitch it at a similar level to an article in a broadsheet newspaper.

Involve your PPI representative in drafting and editing your plain English summary and follow the guidelines below.

**Use these headings**

- What was the problem/question?
- What did we do?
- What did we find?
- What does this mean?

**2. Structure**

1. **Basics**

**5. Useful resources**

- Plain English Campaign [guide to writing medical information](http://www.plainenglish.co.uk/files/medicalguide.pdf)
- INVOLVE [resource](http://www.invo.org.uk/resource-centre/plain-english-summaries/) on plain English summaries
- Digital Curation Centre guide [How to Write a Lay Summary](http://www.dcc.ac.uk/sites/default/files/documents/publications/HowToLaySummariesDec2012.pdf)

Before you finish, check that your summary can be understood by a lay audience

- Ask your PPI representative for feedback
- Check your [Flesch Reading Ease score](http://www.readabilityformulas.com/free-readability-formula-tests.php); higher scores indicate easier reading

**4. Language**

- Using short sentences
- Limiting sentences to one main point

**3. Consider**

- Keep it simple!
- Do not use [jargon](http://www.invo.org.uk/resource-centre/useful-information/jargon-buster/) or scientific terminology
- Use active rather than passive voice
- Use personal pronouns
