# Supplementary material for: Understanding Plain English summaries. A comparison of two approaches to improve the quality of Plain English summaries in research reports
Source: Res Involv Engagem. 2017 Oct 9;3:17. doi: 10.1186/s40900-017-0064-0 (PMC5632836; doi:10.1186/s40900-017-0064-0)
Supplement: Supplementary file 2 — Table of Acronyms. (DOCX 11 kb) [file 40900_2017_64_MOESM2_ESM.docx]

Additional file 2 Table S1: **Acronyms**

| CRUK | Cancer Research UK |
| --- | --- |
| EME | Efficacy and Mechanism Evaluation |
| FRE | Flesch Reading Ease |
| HS&DR | Health Services and Delivery Research |
| HTA | Health Technology Assessment |
| MRC | Medical Research Council |
| NETSCC | NIHR Evaluation Trials and Studies Coordinating Centre |
| NIHR | National Institute for Health Research |
| PES | Plain English Summary |
| PHR | Public Health Research |
